# Primary Mesenteric Smooth Muscle Tumor: An Entity with Unpredictable Biologic Behavior

**DOI:** 10.1155/2013/483689

**Published:** 2013-08-24

**Authors:** Ioannis Kalogiannidis, Thomas Stavrakis, Ioannis Amplianitis, Sophia Grammenou, Georgios Mavromatidis, David Rousso

**Affiliations:** ^1^3rd Department of Obstetrics & Gynecology, Aristotle University of Thessaloniki, Konstantinoupoleos 49, 54642 Thessaloniki, Greece; ^2^Department of Pathological Anatomy, Hippokration General Hospital, Konstantinoupoleos 49, 54642 Thessaloniki, Greece

## Abstract

Smooth muscle tumors of the mesentery are rare lesions with unpredictable, usually malignant, biologic behavior irrespective of their histologic appearance. Such case is presented here. We present a case of a large smooth muscle tumor located in the mesentery of a 48 years old patient. The histopathologic features of the surgically excised tumor were that of a benign-appearing smooth muscle tumor, either a primary mesenteric smooth muscle tumor of unknown biologic behavior or a parasitic leiomyoma. The patient was discharged 4 days after from the hospital without any early postoperative complication. Close followup was further decided. Nine months after her primary therapy, our patient is alive and with no evidence of recurrent disease. Increased awareness must be considered for large mesenteric smooth muscle tumors, because even when they present indolent histologic features, they usually behave aggressively.

## 1. Introduction

Solid primary tumors of mesenteric origin are quite rare. Among them, gastrointestinal stromal tumors (GISTs) and smooth muscle tumors seem to be the most common neoplasms [1–3]. Regarding the latter tumors, especially those of large size, the prediction of the biologic behavior based on histologic grounds is not efficient. Most of the large mesenteric smooth muscle tumors behave aggressively irrespective of their histologic appearance [4–6].

We present a case of a 48 years old female patient with a large smooth muscle tumor located in the mesentery with a synchronous uterine leiomyoma. Diagnostic and therapeutic approach, postoperative dilemmas, and review of the literature are presented as well.

## 2. Case Presentation

**Figure 1:**
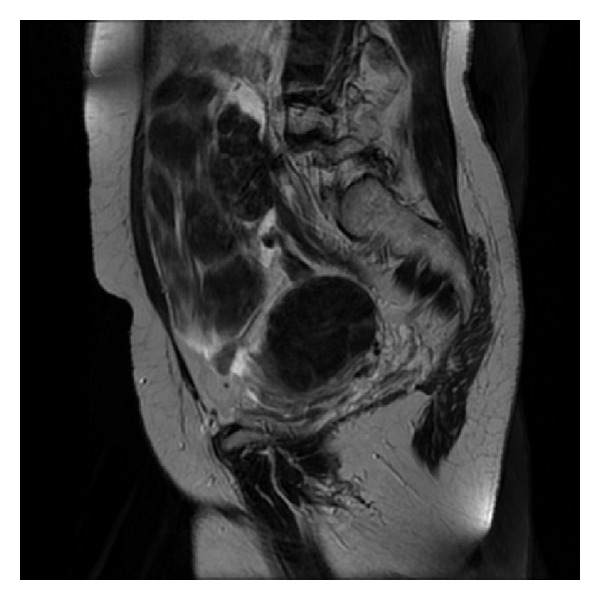
Magnetic resonance imaging (MRI) of the abdomen revealing a solid mass 20 cm of maximum diameter.

A 48 years old woman, mother of two children with normal menstruation and insignificant prior history, presented with abdominal pain. Ultrasound examination and MRI of the abdomen revealed a solid mass, 20 cm of maximum diameter in the pelvis (Figure 1), with no sign of peritoneal or nodal metastasis. Tumor markers were negative. The patient underwent laparotomy, and intraoperative gross inspection confirmed the presence of a solid mass originating from the mesentery. A uterine tumor with maximum diameter of 6 cm was also inspected. The mesenteric tumor was completely excised and was followed by frozen section examination which revealed a spindle cell neoplasm. Nuclear atypia, increased mitotic index, or tumor cell necroses were not observed. Because of the coexistence of the uterine tumor, hysterectomy with bilateral adnexectomy was performed. Infracolic omentectomy was also performed, and peritoneal biopsies were taken. Thorough counseling was made, and informed consent was taken preoperatively from the patient concerning the possibility of hysterectomy.

**Figure 2:**
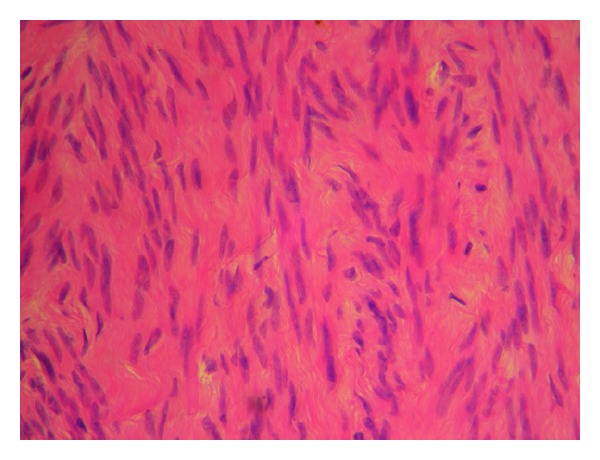
Mesenteric tumor stained with hematoxylin.

**Figure 3:**
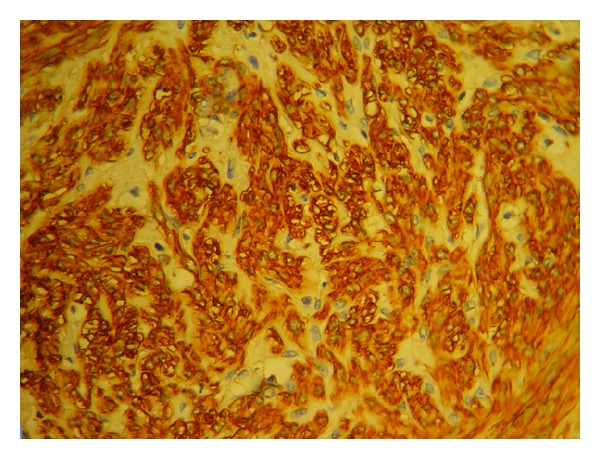
Mesenteric tumor stained with (smooth muscle actin) SMA.

Paraffin-embedded sections of the mesenteric tumor revealed that it was a smooth muscle tumor, composed of anastomosing fascicles of smooth muscle cells with no atypia, mitosis, or tumor cell necrosis. Immunohistochemically, the smooth muscle cells were positive for SMA and desmin and negative for CD117 (Figures 2 and 3). Peritoneal biopsies and omentum were disease free. The uterine tumor was a typical benign leiomyoma.

The patient was discharged four days later. The postoperative course was uneventful. Because of the dilemma regarding the biologic behavior of the mesenteric tumor, long-term close followup was decided. Nine months after surgery, no recurrent disease has been noted.

## 3. Discussion

Primary solid tumors of the mesentery are usually of mesenchymal nature [1, 3]. Most commonly, they are smooth muscle tumors or GISTs [3]. Fibromatosis (desmoid tumor), well-differentiated liposarcoma, malignant fibrous histiocytoma, and peripheral nerve sheath tumors also occur in the location [7, 8].

Regarding the primary, mesenteric smooth muscle tumors, their biologic behavior seems to be unpredictable, because these tumors, when large, usually behave in a malignant fashion, even in the absence of nuclear atypia, tumor cell necrosis, or increased mitotic count [4]. This is in contrast with their uterine counterparts. The uterine smooth muscle tumors with low mitotic count, none-to-mild nuclear atypia, and no tumor cell necrosis are characterized as leiomyomas and behave in a benign fashion [1, 4, 5, 9].

In our case, the histologic examination of the mesenteric tumor showed that it was a smooth muscle tumor with no atypia, no tumor cell necrosis, and no increased mitotic count. If the mesenteric tumor is considered a primary mesenteric smooth muscle tumor, despite the bland histopathologic characteristics, the large size of the tumor indicated that it will behave in a malignant fashion [5, 6, 10].

If the mesenteric tumor is not considered a primary tumor of the mesentery, other entities, mainly the parasitic leiomyoma, should enter the differential diagnosis [5]. The patient had a synchronous uterine leiomyoma, and the possibility of a second leiomyoma detached form a subserosal location and attached to the mesentery could not be excluded. In such a case, the indolent histologic features of the neoplasm assure the benign biologic behavior [5, 11, 12].

Another entity entering the differential diagnosis could be the benign metastasizing leiomyoma, but most of the women suffering from this mysterious condition present lesions in the lungs and have a smooth muscle tumor excised and inadequately studied in the past [12].

In conclusion, primary solid mesenteric tumors constitute a histological heterogeneous group of neoplasms. Histologic examination can reveal the histogenetic nature of a primary solid mesenteric tumor, more often such as GIST, smooth muscle tumor, or desmoid tumor. In the case of the primary mesenteric smooth muscle tumor, the histologic features, namely, the lack of cytologic atypia, mitoses, and tumor cell necrosis do not correlate with the prognosis, because when large, they usually behave in a malignant fashion. On the other hand, a diagnosis of parasitic leiomyoma should be made with great caution. In any case, we believe that mesenteric smooth muscle tumors, either primary or parasitic, regardless of the histologic characteristics, should have close follow up, because of the serious possibility of malignant behavior, even in the absence of histologic criteria of malignancy.
